# Poster Session II – Poster of Distinction II A319 BIOACTIVE PHOSPHOLIPIDS AS MEDIATORS OF ALTERED BEHAVIOUR AND VISCERAL HYPERSENSITIVITY ASSOCIATED WITH MICROBIOTA FROM PATIENTS WITH INFLAMMATORY BOWEL DISEASE (IBD)

**DOI:** 10.1093/jcag/gwaf042.319

**Published:** 2026-02-13

**Authors:** F A Vicentini, J Pujo, M Hall-Bruce, G Rueda, A Nardelli, A Jiang, A Horta, A Khalil, M Quaderi, C Shimbori, E F Verdu, G De Palma, S Collins, P Bercik

**Affiliations:** McMaster University, Hamilton, ON, Canada; McMaster University, Hamilton, ON, Canada; McMaster University, Hamilton, ON, Canada; McMaster University, Hamilton, ON, Canada; McMaster University, Hamilton, ON, Canada; McMaster University, Hamilton, ON, Canada; McMaster University, Hamilton, ON, Canada; McMaster University, Hamilton, ON, Canada; McMaster University, Hamilton, ON, Canada; McMaster University, Hamilton, ON, Canada; McMaster University, Hamilton, ON, Canada; McMaster University, Hamilton, ON, Canada; McMaster University, Hamilton, ON, Canada; McMaster University, Hamilton, ON, Canada

## Abstract

**Background:**

IBD affects globally over 6 million patients, who often suffer from chronic abdominal pain and psychiatric comorbidities that can persist in the absence of colitis. The gut microbiota is linked to IBD, pain and mood disorders, but its role in modulating pain and mental health in IBD remains unclear. Previously, our group have linked microbial phospholipids lysophosphatidylcholine (LPC) and lysophosphatidic acid (LPA) to abdominal pain in irritable bowel syndrome. However, it is unknown whether these molecules play a role in IBD.

**Aims:**

To investigate whether bacteria contribute to visceral hypersensitivity and altered behaviour in IBD via the production of LPC and LPA.

**Methods:**

Fecal samples from healthy controls (HC n = 15) and IBD patients (n = 35; 51% Crohn’s disease) were collected to assess the levels of LPC and LPA, and their molecular species, via mass-spectrometry. Selected samples with high LPC/LPA levels (HC n = 5, IBD n = 11) were used to colonize germ-free C57BL/6 mice (n = 115, both sexes). Affective-related behaviour was assessed by open field, light preference and tail suspension tests. Visceral sensitivity was assessed by visceromotor responses to colorectal distension (CRD). Central neuronal activation was studied by cFos immunostaining. Microbiota were profiled via 16S rRNA gene sequencing.

**Results:**

Fecal levels of LPC and LPA, specifically 16:0, 18:0, and 18:1, were higher in IBD patients, mainly in those with chronic abdominal pain (p < 0.01). Mice with IBD microbiota had higher responses to CRD (p < 0.001), particularly to non-noxious stimuli, suggestive of allodynia. They also had reduced preference for the light compartment (p < 0.001), increased suspended immobility (p < 0.001), with no changes in locomotion, indicating anxiety- and depression-like behaviours. IBD mice showed increased neuronal cFos in the amygdala and hippocampus, regions associated with affective behaviours, including pain. Bacterial profiles differed between IBD and HC samples, both from human and mice. Notably, mice with visceral hypersensitivity had lower abundance of Bacteroidales taxa that contains genes for the catabolism of LPC and LPA.

**Conclusions:**

Microbiota from patients with IBD affect the gut-brain axis by inducing changes in anxiety- and depression-like behaviour and triggering visceral hypersensitivity. Bacterial LPC and LPA may be thus underlying chronic abdominal pain in IBD patients, whose colitis is in remission.

**Funding Agencies:**

CCC, CIHRCCF

